# Supplementary material for: Comparative efficacy of Chinese herbal injections for treating severe pneumonia: A protocol for systematic review and Bayesian network meta-analysis of randomized controlled trials
Source: PLoS One. 2022 May 23;17(5):e0262776. doi: 10.1371/journal.pone.0262776 (PMC9126378; doi:10.1371/journal.pone.0262776)
Supplement: S1 Appendix — (DOCX) [file pone.0262776.s002.docx]

**Attachment: Search Strategy**

PubMed

| Number | Search terms |
| --- | --- |
| #1 | Severe pneumonia[MeSH Terms] |
| #2 | Severe pneumonia[Title/Abstract] |
| #3 | #1 OR #2 |
| #4 | Chinese herbal injection[MeSH Terms] |
| #5 | Chinese herbal injection[Title/Abstract] |
| #6 | Traditional Chinese medicine injection[Title/Abstract] |
| #7 | Traditional Chinese medicine[Title/Abstract] |
| #8 | xuebijing[Title/Abstract] |
| #9 | tanreqing[Title/Abstract] |
| #10 | xiyanping[Title/Abstract] |
| #11 | reduning[Title/Abstract] |
| #12 | shenfu[Title/Abstract] |
| #13 | shenmai[Title/Abstract] |
| #14 | #4 OR #5 OR #6 OR #7 OR #8 OR #9 OR #10 OR #11 OR #12 OR #13 |
| #15 | randomized controlled trial[Publication Type] |
| #16 | controlled clinical trial[Publication Type] |
| #17 | random*[All Fields] |
| #18 | #15 OR #16 OR #17 |
| #19 | #3 AND #14 AND #18 |

Web of Science

| Number | Search terms |
| --- | --- |
| #1 | TOPIC: Severe pneumonia |
| #2 | TOPIC: Chinese herbal injection |
| #3 | TOPIC: Traditional Chinese medicine injection |
| #4 | TOPIC: Traditional Chinese medicine |
| #5 | TOPIC: xuebijing |
| #6 | TOPIC: tanreqing |
| #7 | TOPIC: xiyanping |
| #8 | TOPIC: reduning |
| #9 | TOPIC: shenfu |
| #10 | TOPIC: shenmai |
| #11 | #2 OR #3 OR #4 OR #5 OR #6 OR #7 OR #8 OR #9 OR #10 |
| #12 | #1 AND #11 |

Cochrane Library

| Number | Search terms |
| --- | --- |
| #1 | severe pneumonia:ti,ab,kw |
| #2 | Chinese herbal injection:ti,ab,kw OR Traditional Chinese medicine injection:ti,ab,kw OR Traditional Chinese medicine:ti,ab,kw OR xuebijing:ti,ab,kw OR tanreqing:ti,ab,kw |
| #3 | xiyanping:ti,ab,kw OR reduning:ti,ab,kw OR shenfu:ti,ab,kw OR shenmai:ti,ab,kw |
| #4 | #2 OR #3 |
| #5 | randomized controlled:pt |
| #6 | #1 AND #4 AND #5 |

Embase

| Number | Search term |
| --- | --- |
| #1 | 'severe pneumonia':ti,ab,kw |
| #2 | 'chinese herbal injection':ti,ab,kw OR 'traditional chinese medicine injection':ti,ab,kw OR 'traditional chinese medicine':ti,ab,kw OR xuebijing:ti,ab,kw OR tanreqing:ti,ab,kw OR xiyanping:ti,ab,kw OR reduning:ti,ab,kw OR shenfu:ti,ab,kw OR shenmai:ti,ab,kw |
| #3 | random:ti,ab,kw |
| #4 | #1 AND #2 AND #3 |

CNKI

| Number | Search term |
| --- | --- |
| #1 | (SU='severe pneumonia' OR SU='severe pulmonary infection') AND (SU='injection' OR SU='extractive' OR SU='for injection' OR SU='Xuebijing' OR SU='Tanreqing' OR SU='Reduning' OR SU='Xiyanping' OR SU='Shenfu' OR SU='Shenmai') |

Wanfang Data

| Number | Search term |
| --- | --- |
| #1 | (SU:'severe pneumonia' OR SU:'severe pulmonary infection') AND (SU:'injection' OR SU:'extractive' OR SU:'for injection' OR SU:'Xuebijing' OR SU:'Tanreqing' OR SU:'Reduning' OR SU:'Xiyanping' OR SU:'Shenfu' OR SU:'Shenmai') |

VIP

| Number | Search term |
| --- | --- |
| #1 | (ti,kw='severe pneumonia' OR ti,kw='severe pulmonary infection') AND (ti,kw='injection' OR ti,kw='extractive' OR ti,kw='for injection' OR ti,kw='Xuebijing' OR ti,kw='Tanreqing' OR ti,kw='Reduning' OR ti,kw='Xiyanping' OR ti,kw='Shenfu' OR ti,kw='Shenmai') |
