# Supplementary material for: Comparative efficacy of Chinese herbal injections for treating severe pneumonia: A protocol for systematic review and Bayesian network meta-analysis of randomized controlled trials
Source: PLoS One. 2022 May 23;17(5):e0262776. doi: 10.1371/journal.pone.0262776 (PMC9126378; doi:10.1371/journal.pone.0262776)
Supplement: Attachment — Submitted filename: Response to Reviewers.docx [file pone.0262776.s005.docx]

We would like to express our sincere thanks to the reviewers for the constructive and positive comments.

Replies to Reviewer 1

1. lines 227-228 “…(CAP) whom were not all needed to be…”

Answer: The correction has been made in the revised version and the English writing has been improved carefully.

2. Line 48 Authors focused on CHIs for severe pneumonia. CHIs are mostly used in China, I would suggest add background information about prevalence of pneumonia/ severe pneumonia in China or at least globally rather than just data in US.

Answer: The prevalence of pneumonia/severe pneumonia in China is deficiency. The only data was about the prevalence of pneumonia in 2 weeks recorded in China Health Statistics Yearbook in 2013 and we have added this content in introduction.

3. Line 113 for the primary outcomes. I knew why authors choose clinical effective rate rather than mortality. But I would still say collecting data for mortality is important if this is an important outcome. If no study reported this, this should be clarified and discussed.

Answer: The 28-day mortality has been added in the revised version as one of the primary outcomes. If we find that there are not enough original studies reporting 28-day mortality when we conduct this NMA, we will discuss and clarify this problem.

4. Line 134 under “Study selection and data extraction”:…screen the studies according to the inclusion criteria. The “inclusion criteria” is not accurate as you will refer to both inclusion and exclusion criteria. Consider “eligible criteria”. Same in line 136

Answer: The correction has been made in the revised version.

5. Line 146 “Evaluation of study quality and risk of bias” What is study quality mean? If you use aOB to assess risk of bias then it’s risk of bias. There are still mixing use of study quality and risk of bias in the method. If you used GRADE to assess certainty of evidence (more used in latest GRADE series than “quality of evidence”), then it’s certainty of the evidence. Please refer to https://www.bmj.com/content/371/bmj.m3900.abstract. Also, authors may consider how to draw the conclusion according to the GRADE approach (article in same link).

Answer: The correction has been made in the revised version and the details about how to draw the conclusion according to the GRADE approach have been added in the revised version.

6. Line 175, authors considered statistically significant. Will there any consideration on clinical significant based on minimal important difference (MID)? Better to clarify.

Answer: Minimal important difference (MID) is mainly used to explain the results of scores or scales. However, our primary outcomes are 28-day mortality and clinical effective rate, which do not involve scores or scales. Moreover, we don’t find recognized MID of our outcomes by searching databases. Hence, we don’t consider the clinical significant based on minimal important difference.

7. Lines 221-223 Need citations to support the sentence “a large number of studies…”

Answer: The relevant citations have been added in the revised version.

8. Lines 224-225 The results are yet to come, how could we know CHIs “could be widely used because of its efficacy and safety”? Even when you have data that shows very good benefit in your final NMA, you will still need to consider the certainty of the evidence to give the conclusion cautiously.

Answer: This sentence has been deleted in discussion.

9. Lines 226-228: according to authors’ description, SP is covered by CAP, which could also be seen in the references 28-29. It seems to me that the NMA in 2019 by Huang X et al has already included all the CAP populations and you are selecting a group of patients from the whole bunch. I did not see a high value of this point now.

Answer: Actually, we aim to express that SP is more serious than CAP and therefore the CHIs utilized between them are different. Maybe it is repetitive with the following contents. Hence, we decide to delete these sentences after considering your advice.

10. Lines 236-237 “…proved to be effective by lots of high quality RCTs[15-17, 44]”. What does this “high quality” mean? Have you assessed them already? I would not say any quality thing without any assessment.

Answer: We have deleted “high quality” in this sentence.
